# How Group A Streptococci Hide in Macrophages

**DOI:** 10.1371/journal.pmed.0030095

**Published:** 2006-01-17

In the past 10 years, evidence has suggested that group A streptococci (GAS) adhere to epithelial cells and invade them. In 1994, LaPenta and colleagues found that GAS could invade human epithelial cells, in some cases better than classical intracellular bacterial pathogens such as *Listeria* and *Salmonella* spp. can.

Several studies quickly confirmed this landmark finding and produced further evidence to support this mechanism. One study showed that high-frequency invasion needed expression of M protein (a surface protein, variation in which provides the basis of Lancefield's method of serotyping GAS) and/or fibronectin-binding proteins such as SfbI. Another study found that the M1 serotype of GAS was particularly adept at intracellular invasion of epithelial cells.

It appeared, therefore, that GAS could invade human cells using several mechanisms, which suggested that intracellular invasion played an important role in the disease pathogenesis. The most direct evidence for intracellular invasion came from studies of patients with recurrent tonsillitis, when it was found that antibiotics failed to eradicate streptococci from the throat in about 30% of cases of pharyngotonsillitis. Osterlund and colleagues showed that tonsils excised from such patients contained intracellular GAS.

The reason for invasion of host cells is not clear, although it was suggested that streptococci might find the intracellular environment a good place to avoid host defense mechanisms. Other theories suggested that internalization played a role in the carriage and persistence of streptococci. Internalization might also be involved in the invasion of deeper tissues, although some studies have found that low virulence was associated with internalization.

In this month's *PLoS Medicine*, Pontus Thulin, Anna Norrby-Teglund, and colleagues try to further elucidate the mechanism behind GAS pathogenesis by examining the in vivo interactions between GAS and cells involved in innate immune responses. The team used human biopsies collected from 17 patients with soft-tissue infections. They found that host phagocytic cells, primarily macrophages, were being used as reservoirs for intracellular bacteria during the acute tissue infection.

The data suggest that this could be a mechanism to avoid antibiotic eradication, which might explain why a high bacterial load was present even in tissues collected after prolonged intravenous antibiotic therapy. The authors also note that the localization of GAS varied and depended on the severity of tissue infection; intracellular localization was most frequent in noninflamed tissue, which also had a low bacterial load. Osterlund's study had already shown that intracellular GAS was present in pharyngeal epithelial and macrophage-like cells of patients with tonsillitis. However, the current study by Thulin and colleagues shows for the first time that this occurred in vivo during severe invasive GAS soft-tissue infections in humans, even while patients are on antibiotic therapy.

The theoretical implication of this study is that if intracellular bacteria are most commonly found in newly involved tissue with low inflammation and low bacterial load, it might be the case that internalization could promote the spread of bacteria within the tissue. This so-called Trojan horse approach is also seen in the fish, and sometimes human, pathogen *Streptococcus iniae*, which invades macrophage-like cells in a similar way. Studies are currently exploring whether this also occurs in GAS soft-tissue infections. But in any case, the clinical implication of this finding is that alternate therapies will be required if clinicians are going to improve the morbidity and mortality associated with severe GAS soft-tissue infections.

## 

**Figure:**
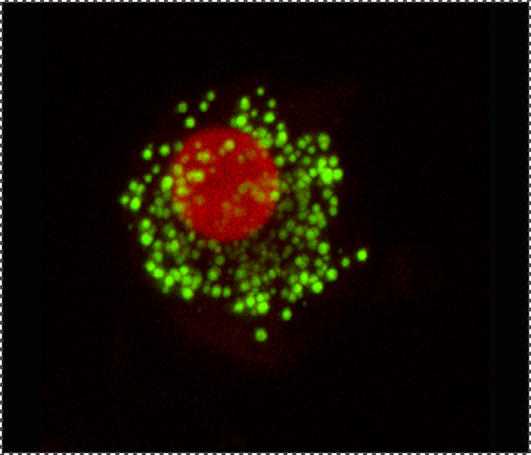
Human macrophages are host cells for intracellular group A streptococci. The figure shows viable (green) bacteria within a macrophage (cell nuclei in red).

